# An expansion to the Nägerl’s theory of proportionality in reduced alveolar bone height models: a 3D finite element analysis

**DOI:** 10.1186/s12903-022-02476-9

**Published:** 2022-10-02

**Authors:** Allahyar Geramy, Amir Reza Geramy, Behzad Fazli

**Affiliations:** 1 Orthodontics Department, Tehran University of Medical Sciences, Tehran, Iran; 2 School of Electrical and Computer Engineering, College of Engineering, University of Tehran, Tehran, Iran

**Keywords:** Finite element method, Center of resistance, Center of rotation, Alveolar bone loss

## Abstract

**Introduction:**

Orthodontic tooth movement is a basic theme in an orthodontic treatment. According to Nägerl, A nearer force application to the center of resistance will bring a farther center of rotation to the center of resistance. The main goal of this study was to evaluate this theory of proportionality by Finite element method (FEM) and find out its applicability in a bone loss situation.

**Materials and methods:**

Three 3D FEM of an upper central incisor were designed in Solidworks 2016, the first one with a healthy bone height (model 1), with 3 mm of bone loss (model 2) and with 4.5 mm of bone loss (model 3). An 0.5 N force was applied in different predefined locations on the labial surface of the crown in model 2. This was 0.2 N for the model 3. The exact location of the center of resistance (Cres), center of rotation (Crot) for each force application point was calculated using a C++ code specially design for this study in all models.

**Results:**

An apical shift of the Cres positions were shown in gradual steps of bone loss from 7.9708 mm incisal from the apex to 6.6292 mm in model 2 and 5.6105 mm in model 3. Modification of the location of the Crot in different force magnitudes and points of force applications were shown whit a constant a*b.

**Conclusion:**

In healthy teeth and teeth with alveolar bone loss, Cres located in the apical third of the root. The product of the distance between the point of force application and Cres (“a”) and the Cres and Crot (“b”) is constant, thus; Nägerl theory is applicable in both healthy and reduced bone height. In this way, applying a single force nearer to the cervical point will result in a more apical location of the Crot, reducing the angle change in the long axis of the tooth.

**Supplementary Information:**

The online version contains supplementary material available at 10.1186/s12903-022-02476-9.

## Introduction

Knowledge of the basic principles of the biomechanics of tooth movement is essential for orthodontic treatment planning [1]. Basically, the stress pattern of the PDL (periodontal ligament) formed by the orthodontic force application is the main predictor of tooth movement. In fact, these stresses cause bone remodeling and subsequent tooth movement [2]. In the area where the PDL is pressed around the tooth, due to the collapse of blood flow and reduced oxygen concentration, a series of enzymatic processes begin in a cascading manner, which is eventually followed by bone resorption in the presence of osteoclasts, Conversely, in the area where the PDL is stretched, these enzymatic processes eventually cause bone deposition in the presence of osteoblasts [3]. The combination of these two processes causes the tooth to move inside the alveolar bone [3]. The center of resistance is considered as a reference point for the predictable movement of the tooth [4]. In fact, the Cres is the point at which, if the force is applied exactly in that direction, the tooth movement will occur without changing the angle of placement inside the alveolar bone. The Crot’s also the point around which the tooth begins to rotate [4]. Accurate determination of the center of resistance and the center of rotation of the tooth can greatly increase the efficiency of the forces applied to the tooth during orthodontic treatment, in general, biomechanically, the dental movements are created by the orthodontic system [4, 5]. This system includes: Force(s) and moment(s). (Either of them may have zero magnitude) Neither of these two components alone can determine the type of tooth movement, but the moment-to-force ratio (M/F) can reliably predict the type of tooth movement. The same displacement of the center of resistance in different dental movements is possible if the force is kept constant. But the center of rotation in dental movements depends on the ratio between the moment to force [6]. On the other hand, the anatomy of the tooth also plays an important role in determining the location of the center of rotation. In fact, a constant ratio of moment to force in a tooth may cause a special type of movement in the other tooth, the same ratio of moment to force shows a different type of tooth movement [6, 7]. According to the theory proposed by Nägerl et al. In 1991, tooth movement based on the assumptions of three-dimensional linear elasticity generally states that in a particular plane, the distance between the point of force application and the center of resistance when multiplied by the distance between the center of resistance and the center of rotation results in a constant value. It is proved to indicate the distribution of forces in the PDL [8, 9]. Early tooth displacement patterns may be affected by variables such as tooth morphology and alveolar bone and periodontal ligament width. It may also occur in adult patients due to periodontal disease, alveolar bone resorption, and some root resorption [10]. This change in the crown-to-root ratio may lead to a change in the biomechanical behavior of the tooth during orthodontic force application, which may be due to the modification of the center of resistance and the center of rotation of the tooth [10, 11]. In this regard, many researchers have tried to determine the exact location of the center of resistance and the center of rotation using various methods such as evaluating two-dimensional, three-dimensional images, connecting electronic and magnetic components to the tooth, using CT scans, etc. The application of different methodologies often does not show good convergent results [1, 12, 13]. The finite element method is widely used in mathematical-physical analysis such as aerospace engineering, fluid mechanics, heat transfer, structural analysis…, and in recent years, the use of this method in a meaningful way in Medical and dental sciences have been expanded. In this way, complex mathematical algorithms are simplified; in fact, complex mechanical models are transformed into small components called elements [14]. For each element, a specific algebraic problem is defined according to the desired conditions and properties, and finally the system solves the problem with the least possible error by integrating all discrete elements [14]. This method has shown its ability to solve clinical problems and issues related to the field of dentistry, including; Initial stress produced in the periodontal membrane by orthodontic loads in the presence of varying loss of alveolar bone [15], evaluation of pressure distribution around dental implants [16], the effect of root length and alveolar bone resorption on dental movements [11, 13], Differences in root stress and strain distribution in buccal and lingual orthodontics [17], Effect of sinus proximity, alveolar bone level, and initial buccolingual inclination on behavior of maxillary first molar under expansion force [18].

The main purpose of this study was to investigate the Nägerl theory in an upper central incisor model with healthy periodontium and in gradual degrees of alveolar bone loss.

## Methodology

**Fig. 1:**
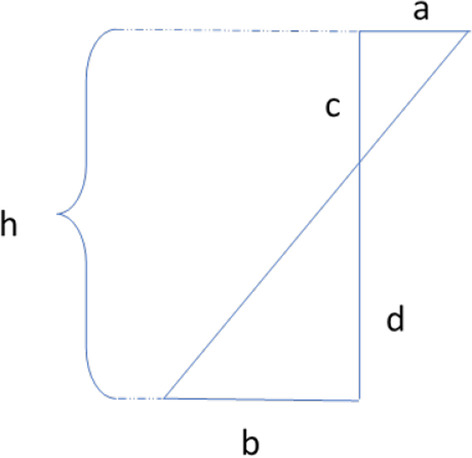
Use the rules of trigonometric ratios for To obtain the Crot and Crest h = the distance between two nodes in the tooth long axis belong to both sides of the sign change ( ±) (= path of nodes) which is a known parameter. “a” and “b” are calculated displacements of the nodes both sides of the sign change

The center of resistance and the center of rotation calculated; when a couple is applied to a restrained body, due to the “moment nature” of the couple, expect the Crot to coincide the Cres and, when a single force is applied, we expect a tipping movement which is believed to be “few millimeters” apical to the Cres. In both situations, a path of nodes was defined between the center of the incisal edge and the apical point. All displacements were recorded relative to this “path of nodes”. We should look for a definite point between the last positive and the first negative one. According to the Fig. 1; h = the distance between two nodes in the tooth long axis belong to both sides of the sign change (±) (= path of nodes) which is a known parameter. “a” and “b” are calculated displacements of the nodes both sides of the sign change.

$$\frac{a}{c} = \frac{b}{d} \to \frac{a}{{b + a}} = \frac{c}{{d + c}}\xrightarrow{{c + d = h}}\frac{a}{{b + a}} = \frac{c}{h}$$

Knowing three elements of this equation, the fourth one is calculated using the below equation:

$$c = \frac{h \cdot a}{{b + a}}$$

Finding “c”; we will be able to find the exact location of the “Cres” and “Crot” of Tipping movement in these conditions.

**Fig. 2:**
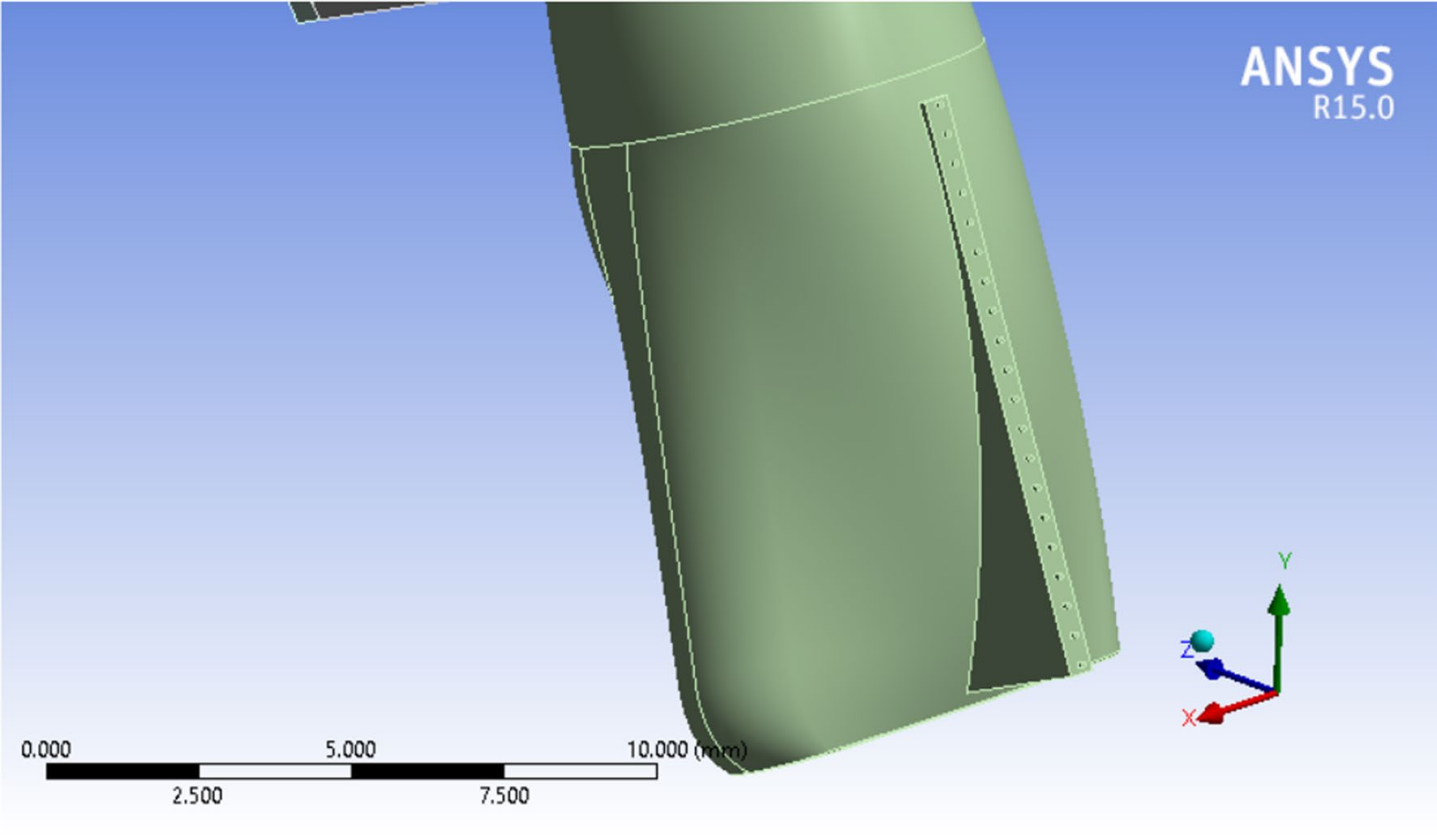
3D model of an upper central incisor with 20 points on its labial surface to be served as different force application sites. X, Y, and Z axes are displayed to show the three-dimensionality of the image, and the dark and light lines indicate the distance of 2500 µm

**Fig. 3:**
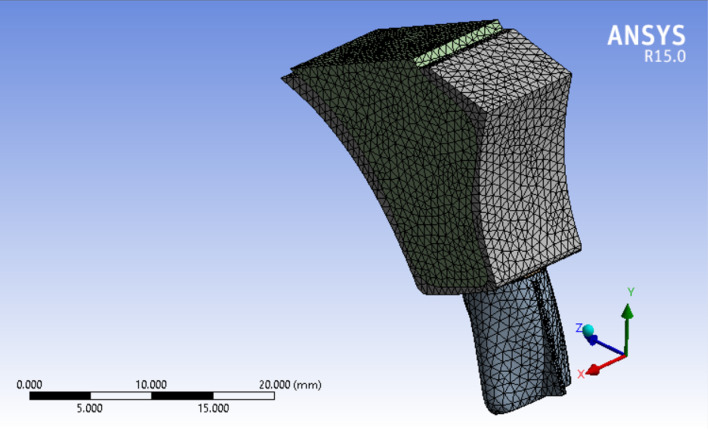
Healthy bone level model. X, Y, and Z axes are displayed to show the three-dimensionality of the image, and the dark and light lines indicate the distance of 5000 µm

**Fig. 4:**
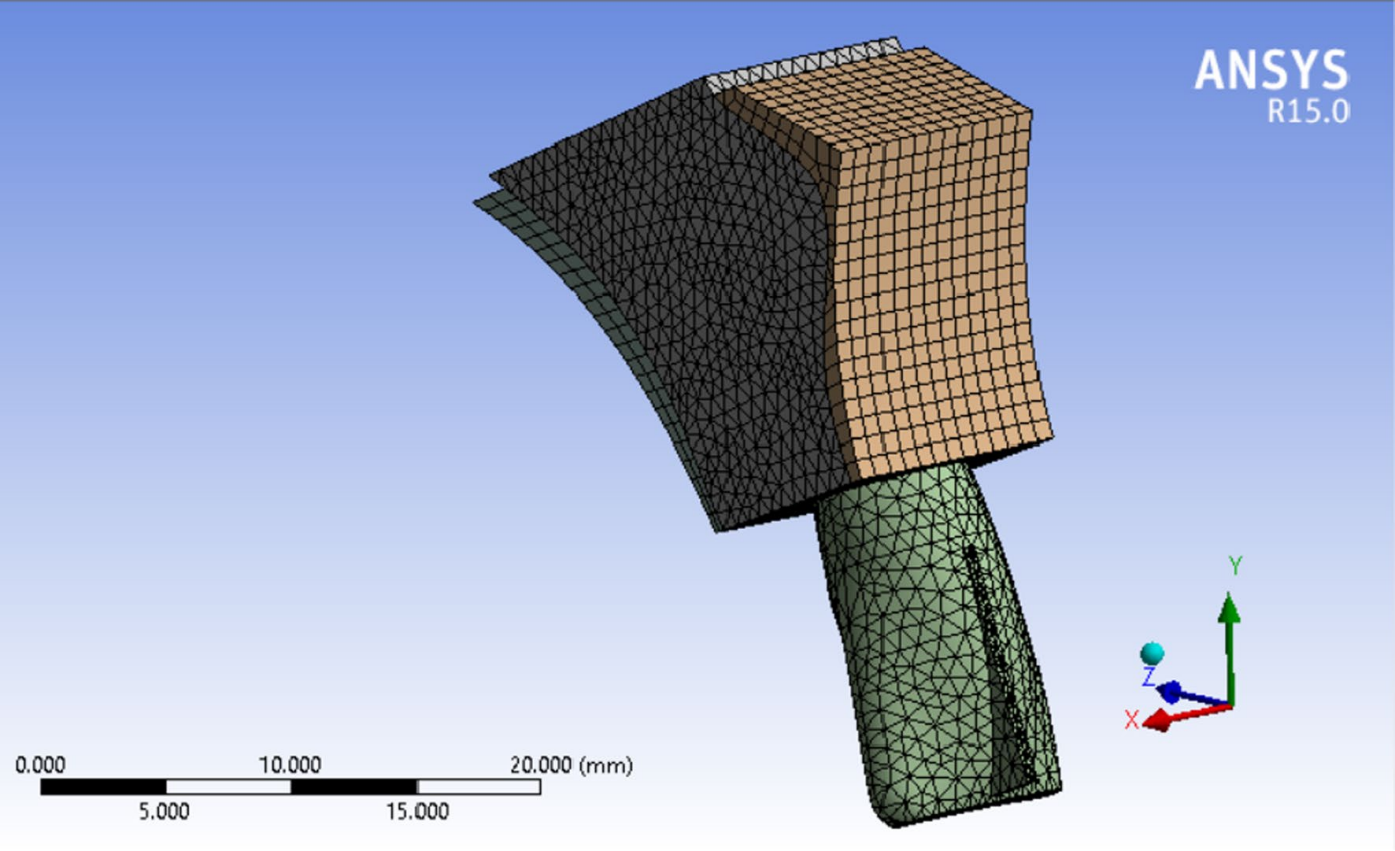
3 mm bone loss model. X, Y, and Z axes are displayed to show the three-dimensionality of the image, and the dark and light lines indicate the distance of 5000 µm

**Fig. 5:**
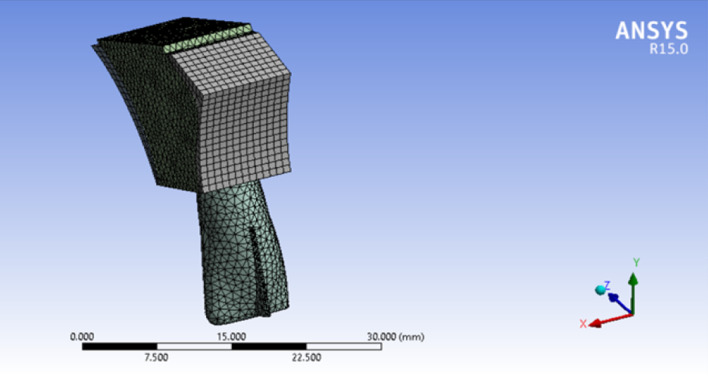
4.5 mm bone loss model. X, Y, and Z axes are displayed to show the three-dimensionality of the image, and the dark and light lines indicate the distance of 7500 µm

Three 3D models of an upper central incisor were designed in Solidworks (Ver. 2021). Figure 2 show the general form of these models with 20 equal distance points on its labial surface to be served as different force application sites. Necessary dimensional information was obtained from Ash dental anatomy with some modifications (Table 1) [17]. All materials are considered to be homogenous (= Homogenous materials have the same density in the volume) and isotropic (= By using isotropic, we mean that all materials have the same Young’s Modulus in three planes of space in three directions). The base of the model was fixed in three planes of space. Periodontal membrane was modeled with a uniform thickness of 0.25 mm around the root surface of the central incisor model. The dental model contains: tooth, PDL, cancellous bone, and dense bone (cortical) The first model (Fig. 3) was in a healthy bone level situation, the second one (Fig. 4) with a 3 mm bone loss (measured from the cervical area) and the third one with a 4.5 mm of bone loss (Fig. 5). The change in the shape of the teeth itself has been largely neglected due to its small size and in order to prevent any kind of symmetry in living environments and in order to create a more complete resemblance to such environments, the methods of creating symmetry of objects in software have not been used in any way [6].

**Table 1:** All materials are considered to be homogenous (= Homogenous materials have the same density in the volume) and isotropic (= By using isotropic, we mean that all materials have the same Young’s Modulus in three planes of space in three directions)

Material	Poisson’s ratio	Young’s modulus (N/mm^2^)
Teeth	0.30	20,300
PDL	0.49	0.667
Cancellous bone	0.38	13,700
Cortical bone	0.26	34,000

**Fig. 6:**
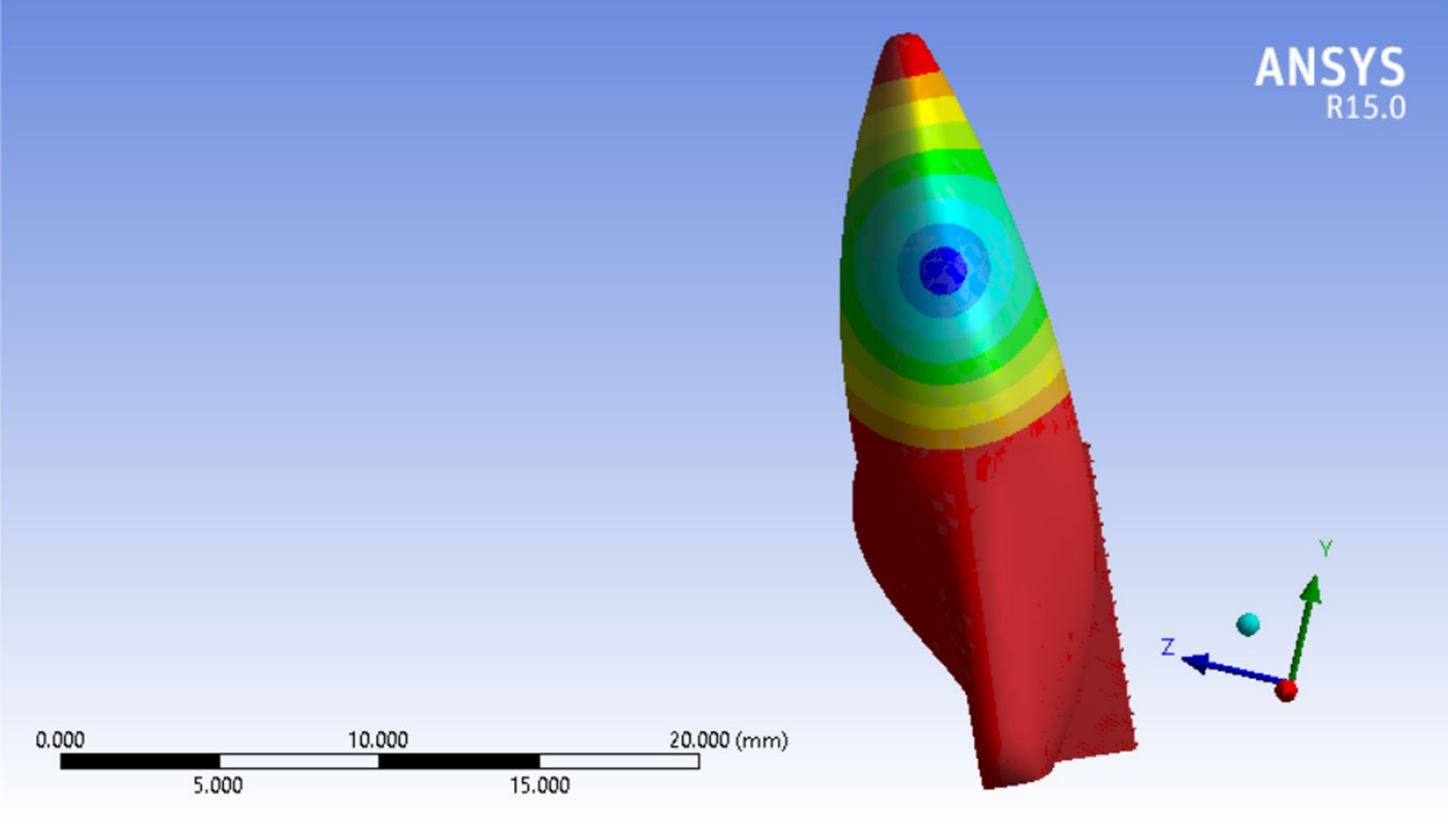
Incisal most point force application. X, Y, and Z axes are displayed to show the three-dimensionality of the image, and the dark and light lines indicate the distance of 5000 µm

**Fig. 7:**
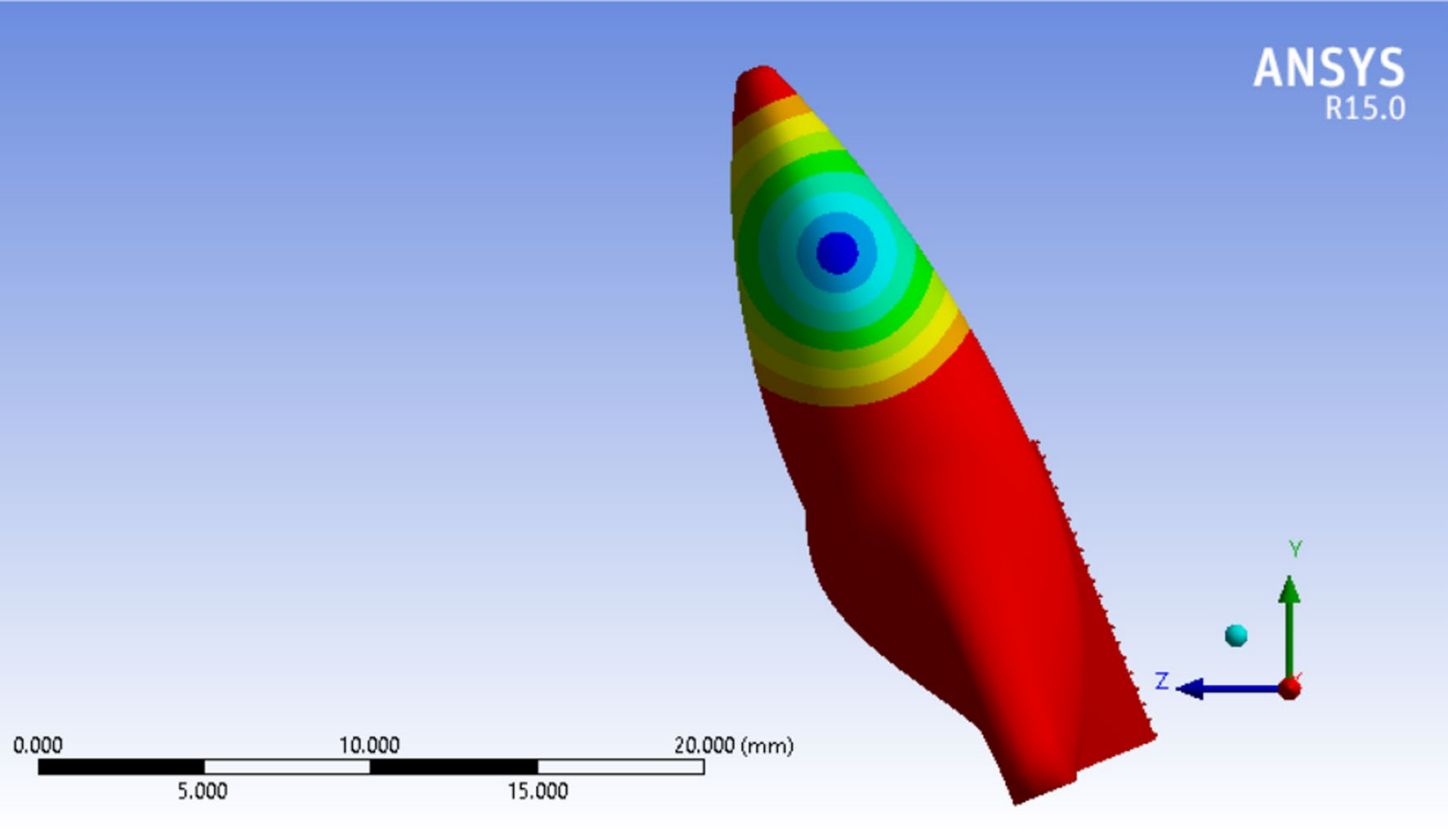
Cervical most point force application. X, Y, and Z axes are displayed to show the three-dimensionality of the image, and the dark and light lines indicate the distance of 5000 µm

An 0.5 N force was applied in a labial-palatal direction in model 1 and 2. In each step of the study to produce tipping with various points of application provided in the modeling phase. In other words, the same force vector was applied in different stages; each stage on a defined point of force application. the same procedure was repeated with a 0.2 N force vector in model 1 and 3. A path of nodes was defined in each model (No. of nodes = 49 in each path) starting from the apex of the tooth ending in the incisal edge. All displacements were detected along the paths in the defined nodes. four large tables (49 line of data for each stage of force application × 20 various forms of tipping which equals 980 data for each table) comparing model 1 and 2 (0.5 N force application) and the same quantity of data in the second comparison (0.2 N force application). A program was implemented by C++ to get the exact location of the center of rotations and resistance (Figs. 6 and 7).

## Results

According to Table 2, the center of resistance is located at a distance of 7.9708 mm from the tooth apex with normal periodontium (model 1) and 6.6292 mm from the tooth apex with 3 mm of alveolar bone loss (model 2). When larger amount of alveolar bone loss (= 4.5 mm) is considered, Cres is located at 5.6105 mm incisal to the apex(model 3); and according to Table 3, in all three models, during the application of force at 20 points, the center of rotation approaches the apex with the cervicalization of the application of force.

**Table 2:** Calculation of the center of resistance the tooth apex in models (Average bold areas) according to the calculations obtained from coding C++

Points	Length of root in heathy model (apicoincisal) [mm]	Length of root in 3 mm alveolar bone loss (apicoincisal) [mm]	4.5 mm alveolar bone loss (apicoincisal) [mm]
1	0	0	0
2	0/48959	0/48959	0/48959
3	0/97919	0/97919	0/97919
4	1/4688	1/4688	1/4688
5	1/9584	1/9584	1/9584
6	2/448	2/448	2/448
7	2/9376	2/9376	2/9376
8	3/4272	3/4272	3/4272
9	3/9167	3/9167	3/9167
10	4/4063	4/4063	4/4063
11	4/8959	4/8959	4/8959
12	5/3855	5/3855	**5/3855**
13	5/8751	5/8751	**5/8751**
14	**6/3647**	6/3647	6/3647
15	**6/8543**	6/8543	6/8543
16	7/3439	7/3439	7/3439
17	7/8335	**7/8335**	7/8335
18	8/3231	**8/3231**	8/3231
19	8/8127	8/8127	8/8127
20	9/3023	9/3023	9/3023

**Table 3:** Calculation of the center of rotations according to the calculations obtained from coding C++

Points	Crot change with healthy model periodontium[mm]	Crot change with 3 mm alveolar bone loss[mm]	Crot change with 4.5 mm alveolar bone loss[mm
1	7/224	6/06572	5/331433
2	7/1882	6/05324	5/323221
3	7/1709	6/03997	5/314111
4	7/1414	6/02582	5/305044
5	7/1746	6/01071	5/295168
6	7/0756	5/99453	5/284657
7	7/0387	5/97718	5/273677
8	6/9987	5/95851	5/261693
9	6/9547	5/93837	5/248852
10	6/907	5/91657	5/234021
11	6/8545	5/89291	5/219729
12	6/7883	5/8671	5/203314
13	6/73142	5/83875	5/185624
14	6/6594	5/80763	5/166573
15	6/5784	5/7733	5/145287
16	6/487	5/73524	5/122104
17	6/383	5/69281	5/096525
18	6/2627	5/6452	5/068013
19	6/1229	5/5914	5/0363
20	5/9585	5/5301	5/000697

**Fig. 8:**
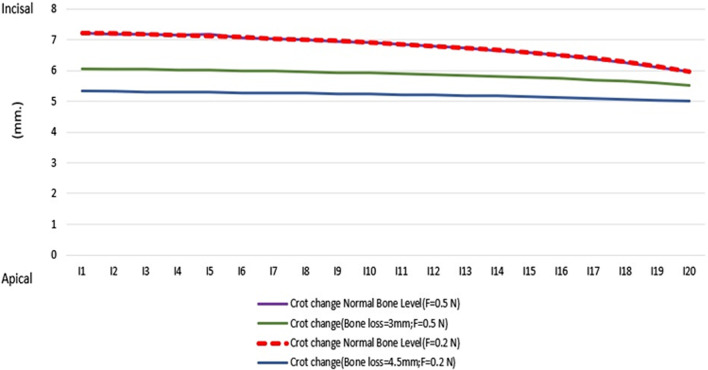
Distance of the center of rotation from the apex to the millimeter at 20 points of force. According to the calculations obtained from coding C++

**Fig. 9:**
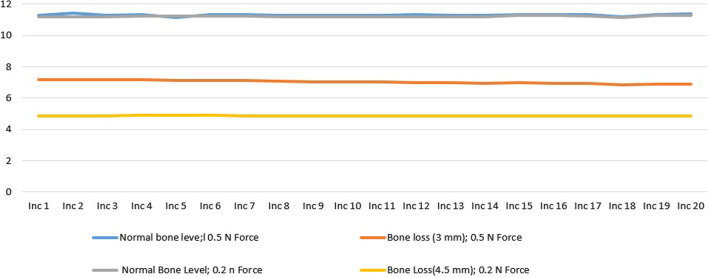
Diagram of changes a * b at 20 points. According to the calculations obtained from coding C++

According to Figs. 8 and 9, changing the amount of force does not play a role in determining the location of the tooth rotation center.

According to Table 4 and to Fig. 9, in the healthy model and the alveolar bone loss models, if the distance of the applied force to the center of resistance is considered as “a” and the distance from the center of rotation to the center of rotation as “b”; the product of these two parameters will be a fixed number.

**Table 4:** Calculate the changes of a * b at 20 points of force in both models according to the calculations obtained from coding C++

Points	Healthy model	3 mm alveolar bone loss model	4.5 mm alveolar bone loss model
1	11/2698	7/1799	4/869363
2	11/4246	7/167	4/871191
3	11/2858	7/1555	4/880694
4	11/3096	7/152	4/885545
5	11/3588	7/136	4/885863
6	11/3116	7/12	4/885827
7	11/3117	7/1037	4/882078
8	11/2759	7/0678	4/868775
9	11/2767	7/0498	4/866708
10	11/2725	7/0315	4/877473
11	11/2686	7/0128	4/866568
12	11/343	6/9939	4/866573
13	11/2658	6/9756	4/864523
14	11/2608	6/9564	4/859327
15	11/3276	6/954	4/882356
16	11/3292	6/9576	4/881485
17	11/3293	6/9407	4/880172
18	11/2103	6/9318	4/840519
19	11/333	6/9036	4/876548
20	11/362	6/8982	4/88221

Assessing the data shows that more cervically applied force vectors cause an apical shift in the location of Crot points. In Figs. 6 and 7, in the tooth with 3 mm alveolar bone loss, the forces are applied in the most incisal and cervical points, respectively, and the location of the center of rotation is shown.

## Discussion

According to the findings of Nägerl et al. Regarding the movement of healthy teeth, the position of the center of rotation depends on the M/F ratio, and most centers of rotation are perceived within a narrow range of M/F at the root site, which is called the critical zone. According to Nägerl, theory of proportionality states that in a healthy tooth, if the distance of the force applied to the center of resistance is “a" and the distance of center of resistance to the center of rotation is “b”, the product of these two will be a constant number $a\times b={\sigma }^{2}$. The larger the σ^2^ corresponding to the wider critical zone and the larger σ^2^ makes it easier to predict the Crot, and the lower the value σ^2^ means the higher the sensitivity to reach the Crot location, in theory if σ^2^ = 0 with no matter where the force is applied, rotation around the Cres will occur except for the exact location of the Cres itself [8, 9, 18]. The present study, by examining and comparing the teeth in the healthy state and in the reduced bone levels states, has reached similar results in this regard, because using the finite element method, it was found that the product of “a” and “b” in an alveolar bone loss, and the healthy tooth, is constant, but numerically the product was always less in alveolar bone loss cases. This may indicate that it will be more sensitive to reach the exact location of the Crot due to the smaller σ^2^ and the reduction of the critical area with alveolar bone loss.

According to Geramy with gradual reduction in the alveolar bone height in a simple tipping movement, the center of rotation and the center of resistance moves toward the apex, but its distance to the alveolar crest Is reduced. He also states that in alveolar bone loss, the distance between the center of resistance and the center of rotation is reduced [13], The present study is in accordance with Geramy [13], Many attempts have been made from the past to determine the center of rotation and the center of resistance of teeth with different approaches.

For example, Burstone et al. [5] Using a non-invasive holographic laser method to study the three-dimensional movement of maxillary incisor teeth under different conditions, in this study, 200 g loads were placed on 10:1 model of the maxillary central incisor. It was found that the center of resistance was at a point one-third of the distance from the alveolar crest to the apex. The centers of rotation as measured experimentally differed from the theoretical estimates based on the two-dimensional model in that they were less sensitive in establishing commonly used centers of rotation [5]. In their experimental model for determining the center of resistance and the center of rotation, Pedersen et al. state that the center of resistance in single forces entering the incisor tooth is 33% of the root length, in which the center of rotation is 0.5 mm more apical than the center of resistance [12]. According to Geramy using a three-dimensional model of an upper incisor tooth to investigate the application of different Force and moment ratios in creating tooth movement and finding the exact center of rotation, He states that the M/F required to create the Bodily movement in the model designed in that study was − 8.44, with the center of rotation occurring at 923.98 mm of the tooth apex. The center of rotation of the simple tipping was 6.53 mm inside the root, and finally, the M/F was between-6.5 and − 7 to create the controlled tipping movement [6]. In line with the above three studies in the present study, in a tooth with a healthy periodontium by applying a single force at 20 points, in terms of quantitative calculations, the center of resistance was about 7.9708 mm of apex (located in the apical third). According to their research on canine teeth, Choy et al. [20] believe that the center of resistance shifts to the apex with alveolar bone loss [19], and according to the present study, the center of resistance moves to the apex with maxillary central incisor with alveolar bone loss. Given the similarity in the results, this theory seems to be true for other teeth in both jaws as well (Additional files 1, 2, 3 and 4).

## Conclusion

According to the findings of this study, the following can be mentioned;

1. The center of resistance is located in the apical third of the root. In both healthy model and reduced bone height level model.
2. With alveolar bone loss, the center of resistance and the center of rotation are both shifted towards the apical area.
3. The product of the distance between the point of force application and Cres (“a”) and the Cres and Crot (“b”) is constant. The smaller “a”s result in larger”b”s. In this way, applying a single force nearer to the cervical point will result in a more apical location of the Crot, reducing the angle change in the long axis of the tooth.
4. The Nägerl theory is shown to be right in both healthy and reduced bone level height model numerically.

## Supplementary Information

**Additional file 1:** 3 mm bone loss model.

**Additional file 2:** 4.5 mm bone loss model.

**Additional file 3:** Coding in C++ (= a computer programming language).

**Additional file 4:** Normal bone height model.

## Data Availability

All data generated or analysed during this study are included in this published article.

## Abbreviations

FEM: Finite element method
Cres: Center of resistance
Crot: Center of rotation
PDL: Periodontal ligament
M/F: Moment-to-force ratio
N: Newton
