# Supplementary material for: An expansion to the Nägerl’s theory of proportionality in reduced alveolar bone height models: a 3D finite element analysis
Source: BMC Oral Health. 2022 Oct 2;22:434. doi: 10.1186/s12903-022-02476-9 (PMC9526930; doi:10.1186/s12903-022-02476-9)
Supplement: Supplementary file 3 — Additional file 3: Coding in C++ (= a computer programming language). [file 12903_2022_2476_MOESM3_ESM.docx]

***Coding in C^++^ (= a computer programming language)***

Coding in C^++^ helped finding the exact location of the “Cres’ and “CRot” by the above mentioned formula.

-------------------------------------------------------------------------------------------------------------------------

#include<iostream>

#include<string>

#include<vector>

#include<sstream>

using namespace std;

const double level_distance = 0.4896;

const int zero = 0;

const int one = 1;

const int two = 2;

long double calculate_sigma_power_two(long double alpha, long double beta){

long double sigma_power_two = alpha * beta;

return sigma_power_two;

}

long double calculate_beta(long double crot, long double cres){

long double beta = cres - crot;

return beta;

}

long double calculate_alpha(long double cres, long double force_app){

long double alpha = force_app - cres;

return alpha;

}

long double calculate_crot(vector<long double>numbers_to_find_crot){

long double a = numbers_to_find_crot[zero];

long double b = numbers_to_find_crot[one];

long double lower_level_height = numbers_to_find_crot[two];

long double divide_ab = a/b;

long double result = level_distance/(divide_ab + 1);

long double crot = lower_level_height - result;

return crot;

}

long double calculate_cres(vector<long double>numbers_to_find_cres){

long double a = numbers_to_find_cres[zero];

long double b = numbers_to_find_cres[one];

long double incisal_level = numbers_to_find_cres[two];

long double divide_ab = a/b;

long double result = level_distance/(divide_ab + 1);

long double cres = incisal_level - result;

return cres;

}

void run(){

vector<long double>numbers_to_find_crot;

vector<long double>numbers_to_find_cres;

long double force_app;

string line;

getline(cin, line);

istringstream ss(line);

string number;

while(ss >> number){

stringstream convert(number);

long double temp = 0;

convert >> temp;

numbers_to_find_crot.push_back(temp);

}

string line_2;

getline(cin, line_2);

istringstream ss_2(line_2);

string number_2;

while(ss_2 >> number_2){

stringstream convert_2(number_2);

long double temp_2 = 0;

convert_2 >> temp_2;

numbers_to_find_cres.push_back(temp_2);

}

long double crot = calculate_crot(numbers_to_find_crot);

long double cres = calculate_cres(numbers_to_find_cres);

cin >> force_app;

long double alpha = calculate_alpha(cres, force_app);

long double beta = calculate_beta(crot, cres);

long double sigma_power_two = calculate_sigma_power_two(alpha, beta);

cout << "alpha is " << alpha << " " << "beta is " << beta << " " << "sigma to the power of two is " << sigma_power_two << endl;

}

int main(){

run();

}

According to this coding in C^++^:

1. “a” and “b” and the lower node height are entered.
2. “a”, “b” and insical node height.
3. “CRot” and “Cres” are calculated.
4. “The Force application point” is entered.
5. Alpha is calculated using Force App. and “Cres”
6. Beta is calculated using “Cres” and “crot”
7. Sigma power 2 is calculated by multiplying “Alpha” and “Beta”
8. Finally, “Alpha”, “Beta” and “Sigma to the power of 2” are printed.
